# A novel somatic mutation in *POLE* exonuclease domain associated with ultra-mutational signature and MMR deficiency in endometrial cancer: a case report

**DOI:** 10.1186/s13000-023-01287-y

**Published:** 2023-02-10

**Authors:** Jiantao Cui, Xiuying Chen, Qian Zhai, Na Chen, Xiaodan Li, Yuli Zhang, Hui Wang, Xin Bian, Na Gao, Deyi Chen, Zhihong Chen, Shibiao Zhang, Yan Chen

**Affiliations:** 1 Department of gynaecology, Cangzhou Hospital of Intergarted TCM-WM, 061000 Cangzhou, Hebei China; 2 Xiamen Spacegen Co.,Ltd, 361100 Xiamen, China

**Keywords:** Endometrial cancer, *POLE* EDM, Hypermutated, TMB

## Abstract

**Background:**

Defect in proofreading exonuclease activity of polymerases epsilon and delta (Pols ε and δ) leads to mutagenesis and genomic instability and has been described in several cancer types. Somatic *POLE* exonuclease domain mutations (EDMs) have been reported in 7–12% endometrial cancers (ECs) and defined a subgroup of endometrial cancers with ultrahigh somatic mutation frequencies, high tumor infiltrated lymphocytes and favorable outcomes.

**Case presentation:**

Herein, we presented a novel somatic mutation in *POLE* exonuclease domain associated with ultra-mutational signature and MMR deficiency in endometrial cancer. A novel *POLE* EDM (p.T278K) was found by a 11-gene NGS panel. The MSS status detected by the MSI test was inconsistent with the dMMR status by IHC. The loss of *MSH6* expression in the tumor could be interpreted by the two nonsense mutations (p.E1234* and p.E1322*) of the *MSH6* gene which may lead to truncated proteins. The T278K mutation was pathogenic identified by a 602-gene NGS panel with 27.3% of C > A substitution, 0.6% of indels, 0.6% of C > G substitution and a high TMB of 203.8 mut/Mb.

**Conclusions:**

We report an endometrial cancer patient harbored a novel somatic *POLE* T278K mutation. This mutation was a novel pathogenic *POLE* EDM should be considered as “*POLE* (ultramutated)” in clinical practice for the molecular classification of EC.

## Background

Defect in proofreading exonuclease activity of polymerases epsilon and delta (Pols ε and δ) leads to mutagenesis and genomic instability and has been described in several cancer types [1, 2]. Germline mutations in the exonuclease domain of Pol ε (*POLE*) and δ (*POLD1*) predispose to colorectal cancer (CRC) and other types of cancer [3, 4]. Somatic *POLE* exonuclease domain mutations (EDMs) have been reported in 7–12% endometrial cancers (ECs) and 1–2% CRC [3–5]. In 2013, the TCGA study identified four molecular subtypes of EC at genomic level using array and sequencing-based data. Among these subtypes, new hotspot mutations in the exonuclease domain of *POLE* defined a subgroup of endometrial cancers with ultrahigh somatic mutation frequencies, high tumor infiltrated lymphocytes and favorable outcomes [5, 6]. This subgroup, termed “*POLE* (ultramutated)”, emerged as a new clinical entity consisting about 7% of ECs. P286R and V411L were the most common reported *POLE* EDMs. However, it is problematic to identify more *POLE* EDMs due to the small sample size in the TCGA study and the special “ultra-mutational” signature. Previous studies have demonstrated that *POLE* EDM ECs are characterized by a high prevalence of C > A substitutions, frequently exceeding 20%; a low proportion of small insertion and deletion mutations (indels); and an extremely high tumor mutational burden (TMB > 100 mut/Mb) [7]. Recently, based on these characteristics, León-Castillo et al. established a scoring system which could be used to evaluate the pathogenicity (*POLE*-score ≥ 4) of a novel *POLE* mutation. Using this scoring system, P286R, V411L, S297F, A456P, S459F, F367S, L424I, M295R, P436R, M444K and D368Y were identified as pathogenic (ultra-mutational signature) *POLE* EDMs. Herein, we presented a novel somatic mutation in *POLE* exonuclease domain associated with ultra-mutational signature and MMR deficiency in endometrial cancer.

## Molecular analyses

Molecular testing using next generation sequencing technology was performed on FFPE tissue obtained through hysterectomy. A H&E-stained section was reviewed by two pathologists to confirm there were over 20% of tumor cells in the tissue specimen. Molecular analyses based on NGS were performed at Xiamen Spacegen Co., Ltd including a 11-gene panel (*POLE*, *TP53*, *PTEN*, *MSH2*, *MSH6*, *MLH1*, *PMS2*, *EPCAM*, *KRAS*, *PIK3CA*, *CTNNB1*) designed for endometrial molecular classification, microsatellite instability (MSI) testing containing 34 loci and a 602-gene panel (2.68 Mb) for tumor mutation burden analysis. DNA was extracted from tissue and peripheral blood samples and quantified for NGS library preparation. For the 11-gene panel, PCR amplicon library was generated using 10 ng of genomic DNA and sequencing at an Illumina MiSeq platform. Raw reads were trimmed and aligned to reference genome (hg19) by Trimmomatic (v0.36) and BWA (v0.7.17). Variant (SNVs and Indels) calling and annotation were performed using Pisces (v5.2.9) and ANNOVAR. For the 602-gene panel and MSI test, hybrid capture library was prepared using 200ng of genomic DNA and sequenced on a MGISEQ-2000RS platform. Sequence alignment, filtering, variant calling and annotation were processed by a bioinformatic pipeline based on BWA(v0.7.17), Samtools (v1.9), GATK (v4.1.7.0), manta (v1.6.0), strelka (v2.9.10) and vep (v106). MSI status was analyzed using MSIsensor-pro (v1.2.0) and classified as high frequency of microsatellite instability (MSI-H, > 30% instable loci) or microsatellite stability (MSS, ≤ 30% instable loci). To evaluate the pathogenicity of the T278K mutation, the proportion of substitutions (C > A, T > G and C > G) and indels and TMB involved in the *POLE* scoring system were assessed by the 602-gene panel sequencing.

## Case presentation

**Fig. 1:**
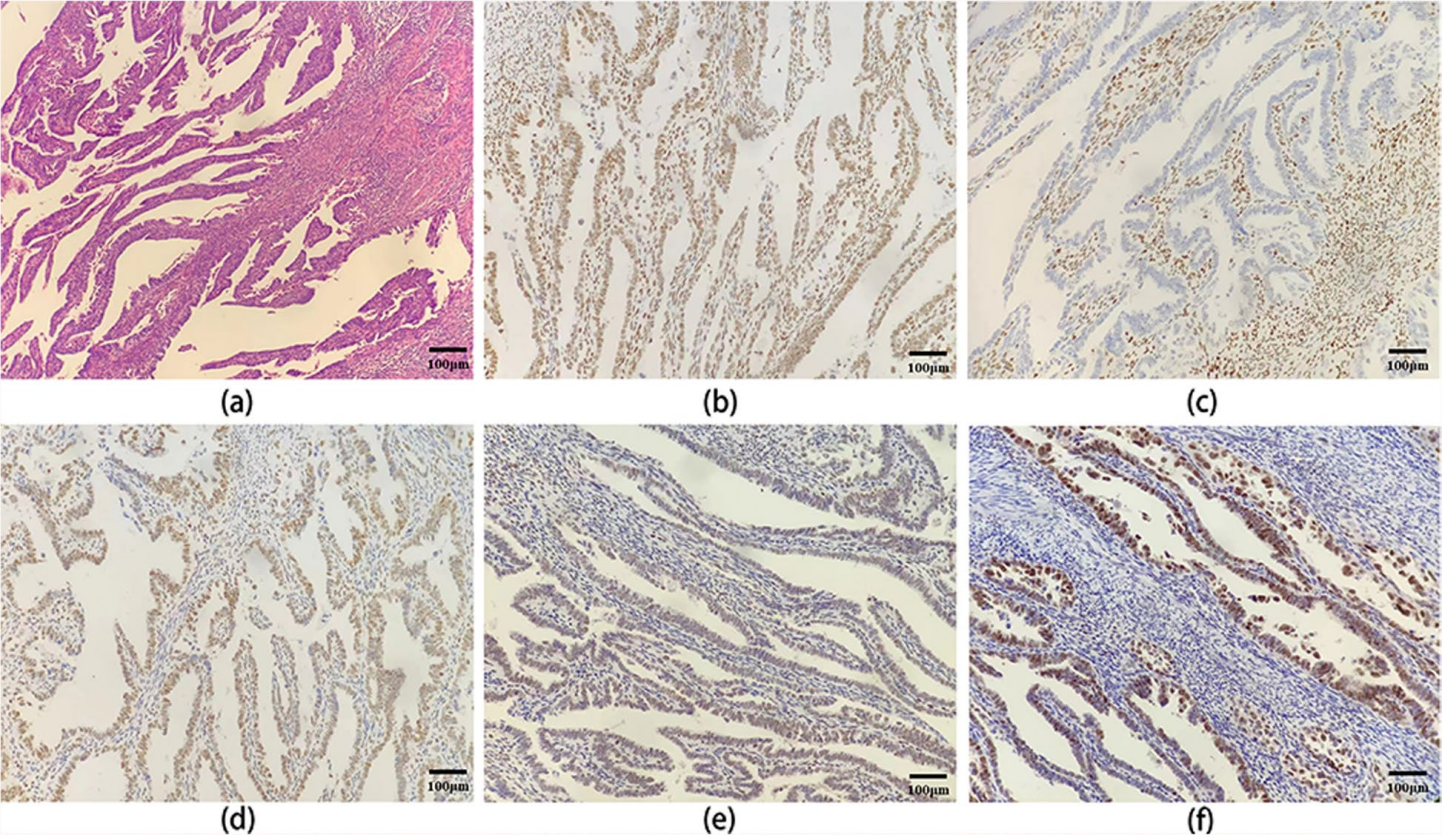
Immunohistochemical stains for MMR proteins (A: MSH2, B: MSH6, C: MLH1, D: PMS2) and p53 protein (F). Magnification: 100

A patient aged 59, premenopausal, with no family history, was diagnosed with endometrial endometrioid adenocarcinoma. She underwent hysterectomy with bilateral salpingo-oophorectomy (TH/BSO) with a tumor mass measuring 4.5 × 3 × 0.8 cm lining in the endometrial cavity, FIGO stage pT1aN0 (stage I) and grade 3. Immunohistochemical (IHC) analysis performed on a LUMATAS automatic pathological staining system (Titan, MXB biotechnologies) showed positive expression of ER(OTI1B1) (60%, intermediate) and PR(OTI2E2) (30%) and negative for HER2(OTI6F3) and PTEN(D4.3). IHC stains for MMR (mismatch repair) proteins showed the tumor was MMR-deficient (dMMR) with loss of MSH6(EP49) protein and normal expression of MLH1(ES05), MSH2(25D12) and PMS2(M0R4G) proteins (Fig. 1b-e). The tumor was intermediate to strong p53 immunostaining (70%) and considered as a wild-type pattern (Fig. 1f).She did not receive any adjuvant therapies after surgery and has shown no recurrence since the initial diagnosis (more than 6 months).

**Fig. 2:**
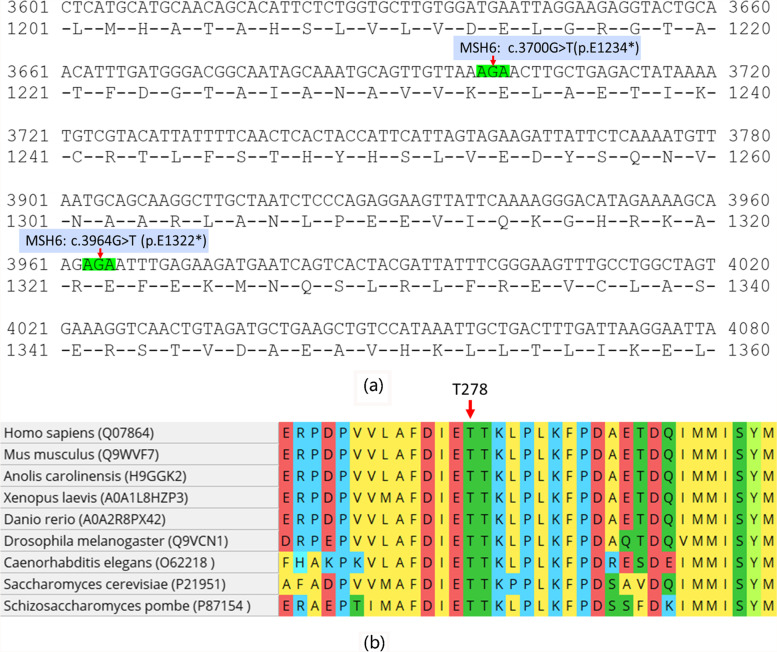
**a** secondary nonsense mutation in MSH6 in AGA (3’ to 5’) context. **b** T278K line in a highly conserved position in the exonuclease domain of POLE.

The 11-gene panel identified a novel *POLE* EDM (c.833 C > A p.T278K, allele frequency 5.49%) with the other seven mutations in the tumor (presented in Table 1) with sequencing quality (Q30) of 87.59% and average depth of 10,628×. The MSS status detected by the MSI test was inconsistent with the dMMR status by IHC. The loss of MSH6 expression in the tumor could be interpreted by the two nonsense mutations (p.E1234* and p.E1322*) of the *MSH6* gene (Table 1) which may lead to truncated proteins. Interestingly, these two mutations were caused by a G > T (or C > A) alteration at an “AGA (or TCT)” context (Fig. 2 A) which may be driven by the *POLE* T278K mutation [8, 9]. This novel mutation occurs at a highly conserved position lining in the exonuclease domain of the *POLE* ε (Fig. 2b). We next investigated the mutational signature of the tumor by a 602-gene NGS panel with blood sample as germline control. The sequencing data quality was 90.42% (Q30) with average depth of 1538×. T278K with the other six mutations found by the 11-gene panel were further identified by the 602-gene panel while one mutation (*MSH6* p.K610E) was conformed a germline variant. The *MSH6* K610E mutation was recorded but not classified in the InSiGHT database (http://insight-database.org/) and considered as a VUS via the ACMG Standards (https://varsome.com/) suggested she was not a Lynch syndrome patient. Furthermore, applied with the *POLE*-scoring system from the León-Castillo’s study, we confirmed that the T278K mutation in this tumor was pathogenic and had a *POLE*-score of 4 with 27.3% of C > A substitution, 0.6% of indels, 0.6% of C > G substitution and a high TMB of 203.8 mut/Mb (Table 2). Additionally, at the same position, a missense mutation (T278M) has been reported in previous studies and annotated as variant of uncertain significance (VUS) with a *POLE*-score of 3 in the study of León-Castillo et al [10, 11]. We evaluated the functional effect of these two mutations using in silico prediction tools: Mutation taster, SIFT, PROVEAN, PolyPhen-2, PANTHER and SNAP2. Both mutations were predicted to be damaging by all the tools (Table 3). In summary, these findings suggest that the T278K mutation is a novel pathogenic *POLE* EDM in EC.

**Table 1:** Mutations in the tumor tissue identified by the 11-gene NGS panel

Gene	cHGVS	pHGVS	Frequency	ClinVar ID	Cosmic ID
*MSH6*	c.1828 A > G	p.K610E	47.99%	rs1172760455	No record
*MSH6*	c.3293G > A	p.C1098Y	5.65%	rs876660564	COSM6956245
*MSH6*	c.3700G > T	p.E1234*	5.52%	No record	COSM1021305
*MSH6*	c.3964G > T	p.E1322*	6.16%	No record	COSM288679
*KRAS*	c.35G > T	p.G12V	6.48%	rs121913529	COSM520
*POLE*	c.833 C > A	p.T278K	5.49%	No record	No record
*TP53*	c.607G > A	p.V203M	4.83%	rs730882003	COSM43599
*TP53*	c.604 C > T	p.R202C	4.37%	rs587780072	COSM46074

**Table 2:** Pathogenicity evaluation based on the *POLE*-score system

Mutation analysis	Results	*POLE*-score
C > A over 20%	27.5% (76)	1
T > G over 4%	3.99% (11)	0
Indels below 5%	0.36% (1)	1
C > G below 0.6%	0.36% (1)	1
TMB over 100 muts/Mb	203.8	1
Recurrent variant in EC	no	0
Total *POLE*-score	4	

**Table 3:** In silico predicting results for the two variants

	*POLE* p.T278K	*POLE* p.T278M
***POLE*** **-score**	4	3
**Mutation taster**	Disease causing	Disease causing
**PolyPhen-2**	Probably damaging (score = 1.000)	Probably damaging (score = 1.000)
**PROVEAN**	Deleterious (score=-5.29)	Deleterious (score=-5.23)
**PANTHER**	Probably damaging	Probably damaging
**SNAP2**	Effect	Effect
**SIFT**	Damaging (score = 0.001)	Damaging (score = 0.001)

## Discussion

To our knowledge, this is the first reported case of endometrial cancer with ultra-mutational signature caused by a novel somatic *POLE* T278K mutation. However, *POLE* T278K as a driven mutation in other type of cancers has been reported in two previous studies. One previous study has reported a somatic *POLE* T278K mutation in a 47-year-old CRC case with MSS and TMB-high (145 mut/Mb) [12]. This case carried multiple germline and somatic variant of uncertain significance in POL genes (*POLE*, *POLD1* and *POLH*) and their pathogenicity was not elucidated [12]. In addition, a study has reported that the germline T278K mutation showing a highly penetrant and autosomal dominant inheritance pattern in a family and was associated with familial polyposis, CRC and extracolonic tumors [13]. All but one of the tumors showed a high TMB (> 10 mut/Mb). Strikingly, among these tumors, one breast tumor showed both an ultra-mutational signature and MMR deficiency resulting from the germline *POLE* T278K mutation and the secondary somatic MMR mutations. Similarly, in this report, we presented a case of EC whose tumor was ultra-mutated and dMMR with MSH6 loss may be driven by the somatic *POLE* T278K mutation and the secondary somatic *MSH6* mutations (E1234* and E1322*). Moreover, we found that both the two nonsense mutations in *MSH6* were caused by a G > T transversion in AGA context, which was a representative characteristic of pathogenic *POLE* EDMs related cancers as confirmed in several previous studies [8, 9, 14]. The ratio of C > A or G > T transversions was included in the *POLE* scoring system and not considered whether these transversions in the TCT or AGA sequence context. Our case together with previous studies suggest that these special transversions may be useful for the identification of novel pathogenic *POLE* EDMs. [8, 9, 14]. *POLE* EDM combined with dMMR or MSI-H was rare in ultra-mutated ECs as previous reported [14]. However, the effect of *POLE* EDMs induced mutagenesis on MMR function has been described in several studies [9–15]. In our case, MSH6 loss may be driven by *POLE* T278K induced secondary nonsense mutations. A remarkable phenotype of the tumor was the MSS status with none of the 34 loci showed instability which may partially due to the tumor was not driven by dMMR.

In summary, we report an endometrial cancer patient harbored a novel somatic *POLE* T278K mutation. This mutation was a novel pathogenic *POLE* EDM identified by the *POLE* scoring system with high (TMB > 100 mut/Mb). The T278K mutation should be considered as “*POLE* ultramutated” in clinical practice for the molecular classification of EC. The tumor also present MMR deficiency with MSH6 loss inconsistent with the MSS status which may be a secondary event induced by the novel pathogenic *POLE* T278K mutation.

## Data Availability

The datasets used and/or analysed during the current study are available from the corresponding author on reasonable request.

## References

[1] Rayner E, Van Gool IC, Palles C (2016). A panoply of errors: polymerase proofreading domain mutations in cancer[J]. Nat Rev Cancer.

[2] Castellucci E, He T, Goldstein DY (2017). DNA Polymerase? Deficiency Leading to an UltramutatorPhenotype: A Novel Clinically Relevant Entity[J]. The oncologist.

[3] Church DN, Briggs SEW, Palles C (2013). DNA polymerase ɛ and δ exonuclease domain mutations in endometrial cancer[J]. Hum Mol Genet.

[4] Palles C, Cazier JB, Howarth KM (2013). Germline mutations affecting the proofreading domains of POLE and POLD1 predispose to colorectal adenomas and carcinomas. Nat Genet.

[5] Levine DA (2013). Integrated genomic characterization of endometrial carcinoma. Nature.

[6] Bellone S, Bignotti E, Lonardi S (2017). Polymerase ε (POLE) ultra-mutation in uterine tumors correlates with T lymphocyte infiltration and increased resistance to platinum-based chemotherapy in vitro. Gynecol Oncol.

[7] León-Castillo A, Britton H, McConechy MK (2020). Interpretation of somatic POLE mutations in endometrial carcinoma. J Pathol.

[8] Shlien A, Campbell BB, De Borja R (2015). Combined hereditary and somatic mutations of replication error repair genes result in rapid onset of ultra-hypermutated cancers. Nat Genet.

[9] Hodel KP, Sun MJS, Ungerleider N (2020). POLE mutation spectra are shaped by the mutant allele identity, its abundance, and mismatch repair status. Mol Cell.

[10] Meng B, Hoang LN, McIntyre JB (2014). POLE exonuclease domain mutation predicts long progression-free survival in grade 3 endometrioid carcinoma of the endometrium. Gynecol Oncol.

[11] Li Y, He Q, Li S (2020). POLE mutation characteristics in a chinese cohort with endometrial carcinoma. OncoTargets and therapy.

[12] Ying J, Yang L, Yin JC, et al. Additive effects of variants of unknown significance in replication repair-associated DNA polymerase genes on mutational burden and prognosis across diverse cancers. J Immunother Cancer. 2021;9(9). 10.1136/jitc-2021-002336. 10.1136/jitc-2021-002336 PMC8420654 34479923

[13] Castellsagué E, Li R, Aligue R (2019). Novel POLE pathogenic germline variant in a family with multiple primary tumors results in distinct mutational signatures. Hum Mutat.

[14] Shinbrot E, Henninger EE, Weinhold N (2014). Exonuclease mutations in DNA polymerase epsilon reveal replication strand specific mutation patterns and human origins of replication. Genome Res.

[15] Yu S, Sun Z, Zong L, et al. Clinicopathological and molecular characterization of high-grade endometrial carcinoma with POLE mutation: a single center study. J Gynecologic Oncol. 2022;33(3). 10.3802/jgo.2022.33.e38. 10.3802/jgo.2022.33.e38 PMC9024187 35320887

